# Mapping the movement for climate change and health in England: a descriptive review and theory of change analysis

**DOI:** 10.1177/17579139211058303

**Published:** 2021-11-24

**Authors:** R Issa, C Baker, R Spooner, R Abrams, A Gopfert, M Evans, G Aitchison

**Affiliations:** Department for Global Health, University College London, London WC1N 1EH, UK; University Hospitals Sussex, Brighton, UK; Centre for Sustainable Healthcare, Oxford, UK; Medact, London, UK; Torbay Council, Torquay, UK; Centre for Environmental Health and Sustainability, University of Leicester, Leicester, UK; Politics and International Studies, Loughborough University, Loughborough, UK

**Keywords:** climate change, sustainability, health, theory of change, social movements, policy change

## Abstract

**Aims:**

There are a growing number of organisations working to address the connections between climate change and health. This article introduces the concept of ‘theories of change’ – the methodology by which organisations or movements hope to bring about social change – and applies it to the current climate change and health movement in England. Through movement mapping, the article describes and offers reflections on the climate change and health ecosystems in England.

**Methods:**

Organisations working on climate change and health in England were identified and publicly available information was collated to map movement characteristics, target stakeholders and methodologies deployed, using an inductive, iterative approach.

**Results:**

A total of 98 organisations working on health and climate change (and/or sustainability) were initially identified, of which 70 met the inclusion criteria. Most organisations target two or more stakeholders, with healthcare workers, management structures, and government being most commonly cited. Methodological approaches identified include Formal education programmes; Awareness-raising; Purchasing-procurement power; Advocacy; Financial; Media-messaging; Networking; Knowledge generation; and Policy making, of which education, awareness-raising, and advocacy are most commonly used.

**Conclusion:**

There is a tendency for climate change and health organisations in England to focus on individual level and sectoral change over system change. More could be made of the potential for the healthcare professions’ voice and messaging for the wider climate movement. Given the rapid boom of climate change and health organisations in recent years, a mind-set shift that recognises different players as part of a cohesive ecosystem with better coordination and collaboration may reduce unnecessary work, and facilitate more cohesive outcomes.

## Introduction

The UCL-Lancet commission on climate change and health was published a decade ago,^1^ but despite its warning that climate change was the greatest threat to health of the 21^st^ century, the UK government has only achieved 2 of the 31 milestones set out in the Progress Report by the Climate Change Commission, and as of 2018 was ‘off track to meet its own emissions targets in the 2020s and 2030s’.^2^ Globally, the story is no brighter. Countries are far from meeting the targets set out by the 2015 legally binding intergovernmental Paris Agreement, and predictions point towards a rise in global temperatures of greater than 1.5 degrees Celsius by the middle of the century.^3^

Awareness and concern regarding climate change – once the domain of climate scientists and fringe groups – has moved into public consciousness, in line with the rise of movements like ‘School Strike for Climate’ and ‘Extinction Rebellion’. In parallel, the medical community has been working through a variety of institutions and methodologies to push for measures to mitigate the climate crisis’ impact on health and the health sector’s contribution to the crisis. In a seeming acknowledgement of these concerns, in 2019 the British government declared a climate emergency, and in October 2020, the Greener NHS England Programme published a target of achieving net-zero by 2040. However, achieving these aims requires ongoing action.

This article introduces the concept of ‘theories of change’ (Box 1) – the methodology by which organisations or movements hope to bring about social change – and applies it to the current climate change and health movement in England. Through movement mapping, this article describes and offers reflections on the climate change and health ecosystems in England.

**Box 1:** 

**Theory of change terminology**
*Theory of change*: Individuals and institutions have beliefs and assumptions about how change happens. These beliefs determine who an organisation chooses to influence, the methods that will be deployed to achieve that influence, and the desired outcome. Such beliefs may be conscious or subconscious. These worldviews are ‘theories of change’,^4^ which, when clearly articulated, can clarify expectations, facilitate better planning, and help map change-points within a broader ecosystem. *Movement*: Groupings of individuals or organisations that focus on specific social or political issues with an aim to carry out, resist, or undo a social change.^5^ *System*: An interconnected set of elements coherently organised so that it achieves something; more than the sum of its parts and defined by complexity arising through relationships and feedback loops among the many elements. When applied to political change, the socio-political organisation of a society, including law and public policy as well as economic and social structures.^4^ *Advocacy*: The process of representing, promoting, or defending a person(s) or cause’s interest or opinion. Policy advocacy is the process of negotiating and mediating a dialogue through which influential networks and decision makers take on ideas and subsequently act upon them.^6^

## Methods

Organisations currently working on addressing health and climate change (and/or sustainability) were identified through the authors’ prior knowledge and expanded by (1) crowd-sourcing submissions and recommendations for organisations through climate and health networks on Twitter,^7^ and (2) Google and Ecosia search engines using keywords, *‘Climate change’ or ‘Sustainability’ and Health*. Organisations were defined according to the criteria in Box 2. Publicly available organisational information was inputted into an online spreadsheet and reviewed by two authors, with disagreements reviewed by a third author. The information included in the spreadsheet was designed to help meet the study aims and included year founded, website, organisational size, membership demographics (if applicable), target stakeholder(s), methodology, and organisational aims. Ethical approval was not required as the study utilises data in the public domain.

**Box 2:** 

**Inclusion and exclusion criteria**
*Inclusion criteria* Organisations, groups, or networks of two or more people with an online presence Currently (wholly or in part) working on the relationship between climate change and/or sustainability, and health Organisational aims can be found online, or are provided on approaching the organisation Operating in England *Exclusion criteria* Climate change and health are not a key part of the organisational or campaign aims Groups with no online presence Group with no clear aims Not explicitly (in part or wholly) working to impact climate change or sustainability, even when an organisations work will have indirect impact on these issues (e.g. advocating for plant-based diets for health benefits only) Groups which have completed a project(s) on Climate Change and Health, and not currently undertaking further work Organisations based outside of England, including those based in and focusing solely on the other nations of the UK *Note that groups using similar methodologies within similar institutions have been combined for the purposes of analysis, including NHS Trust-based advocacy groups, and groups working through the Royal Colleges, and higher education institutions.*

A framework outlining the health system in England and change pathways for climate change as it relates to and interacts with health was developed based on the structure of NHS England and the author’s experiences of working in climate change and health advocacy, and an inductive and iterative approach was taken when defining and mapping the categories of the methodological approaches and stakeholders targeted by different organisations. An inductive approach was chosen because it can help elicit new themes, frameworks, and unexpected findings in a relatively understudied area.^8^

## Results

**Figure 1:**
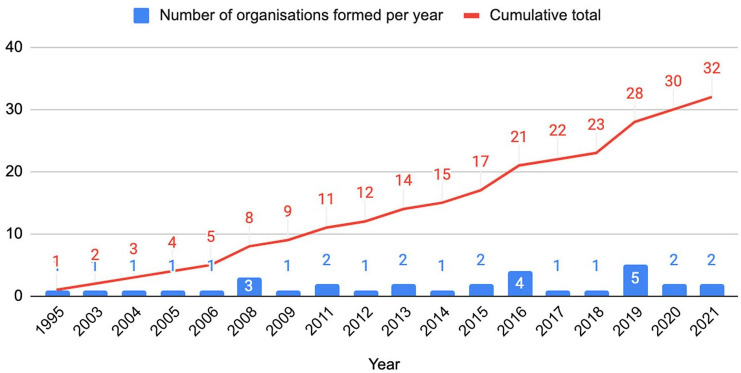
Organisations working on climate change and health, by year founded

A total of 98 organisations working on health and climate change (and/or sustainability) were initially identified, of which 70 met the inclusion criteria. Once similar groups had been combined – (1) NHS Trust-based advocacy groups, (2) groups working through the Royal Colleges, and (3) higher education institutions – 32 groups remained for analysis. There is a steady increase in the number of organisations founded (Figure 1).

**Figure 2:**
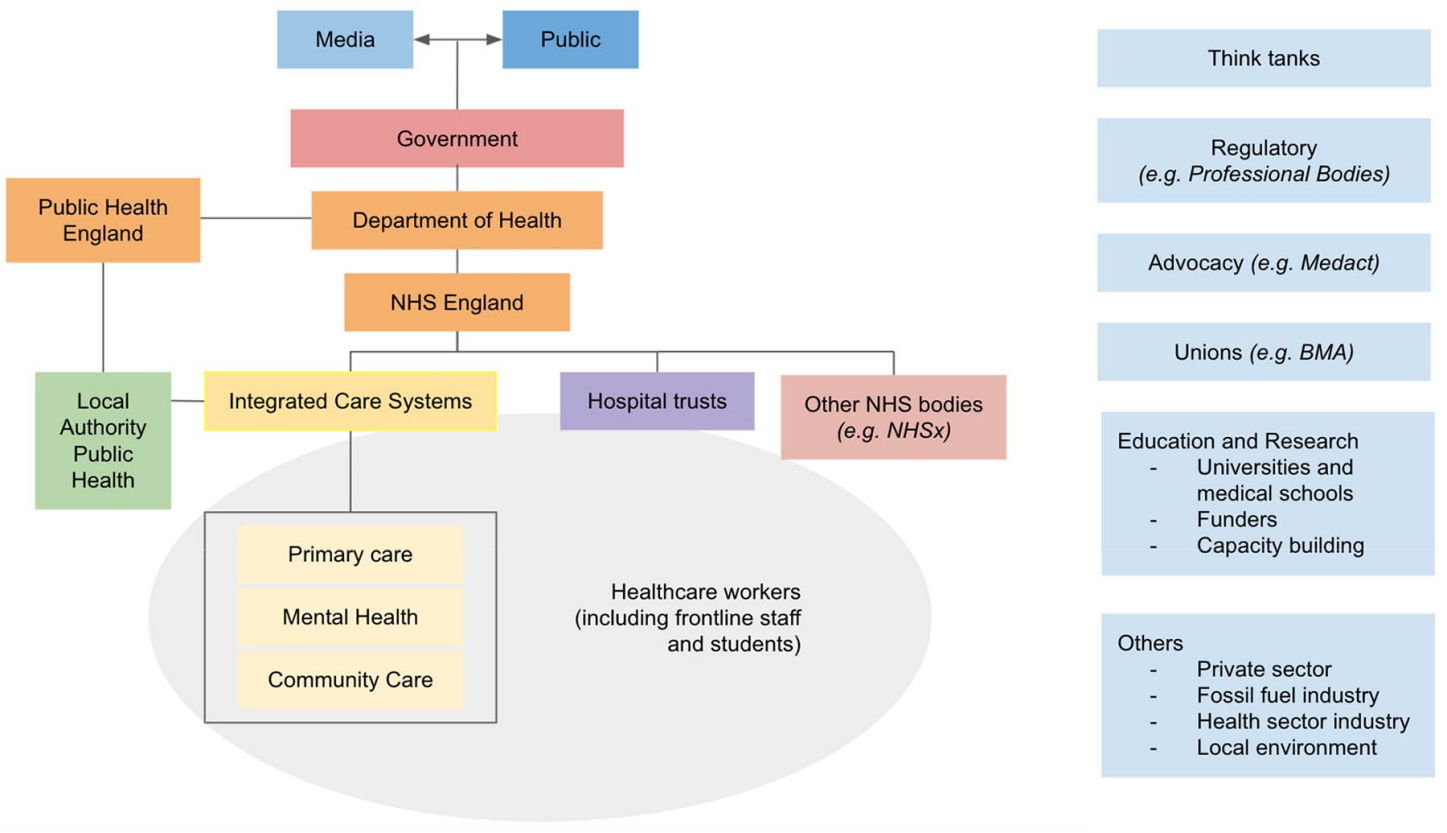
Stakeholder map for the climate change and health space in England At the time of writing, the public health landscape in England is undergoing significant change with the announcement that Public Health England will be removed and its roles divided between existing and new organisations. In particular, the new UK Health Security Agency will take over responsibility for public health protection and infectious disease capability across the UK. Note: Professional bodies are interchangeably referred to as ‘Royal Colleges’ in the text.

**Figure 3:**
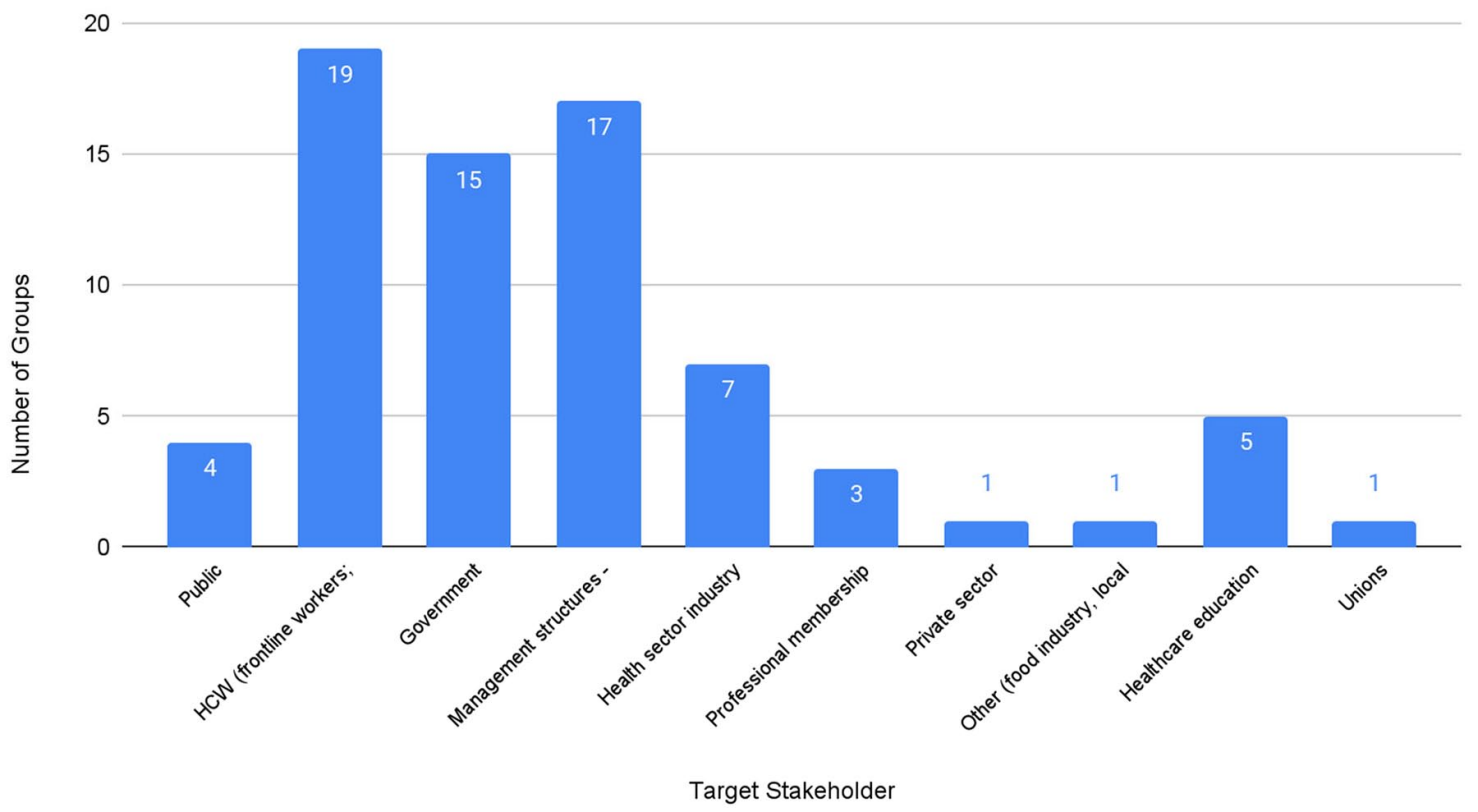
Target stakeholders for organisations working on climate change and health in England

Identified target stakeholders and their relationships are mapped in Figure 2. Of the organisations analysed, most target two or more stakeholders. A total of 19 organisations included healthcare workers among their targets, with 17 organisations targeting management structures (Trusts, Clinical Commisioning Groups (CCGs) replaced by Integrated Care Systems), and 15 organisations aiming to influence Government (Figure 3).

**Figure 4:**
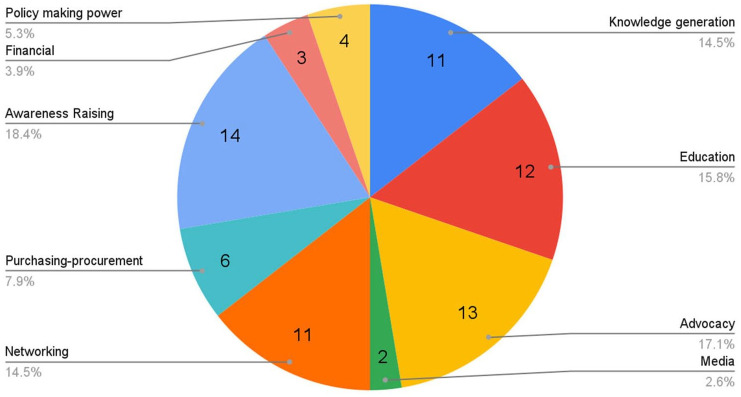
Organisational methodology to impact climate change and health

Groups employ a variety of different methods in order to achieve their impact on the target stakeholders (Box 3), with up to four different methods being used by each organisation. Most frequently used methods included ‘Awareness Raising’ (14), ‘Advocacy’ (13), and ‘Education’ (12) (Figure 4).

**Box 3:** 

**Methodological approaches used by the organisations identified**
**Formal education programmes:** such as courses offered by the *Centre for Sustainable Healthcare*, or conferences, aimed at increasing healthcare workers and students’ knowledge and practice of sustainable healthcare. **Awareness-raising:** activities aimed at increasing awareness of climate change, its impacts on health, and sustainable practice; targeted at individuals with the assumption that increased awareness will lead to behaviour change. **Purchasing-procurement power:** changing the medicines, devices, and equipment purchased by individuals or a health institution to be more sustainable/ecological – for example, reducing single-use plastic items. **Advocacy** **Declare climate emergency:** a symbolic action whereby institutions can publicly declare that there is a climate emergency (+/- commit to measures in response) **Direct communication with policy makers:** using negotiation and other ‘soft power’ skills to influence the creation and development of public policy. **Financial pressure**: seeking change by exerting economic pressure on institutions or systems – for example, divestment or boycott. **Media-messaging:** using the media as an advocacy tool and/or public health framing to influence public opinion, with the overall aim of policy change. **Networking:** connecting individuals, groups, and causes to build collaborations and momentum. **Knowledge generation:** research, evidence-finding, and policy generation – generally conducted by research and educational institutions, and think tanks. **Policy making:** the development and introduction of new policies by policy-making bodies, such as the government or the Department of Health.

## Discussion

**Figure 5:**
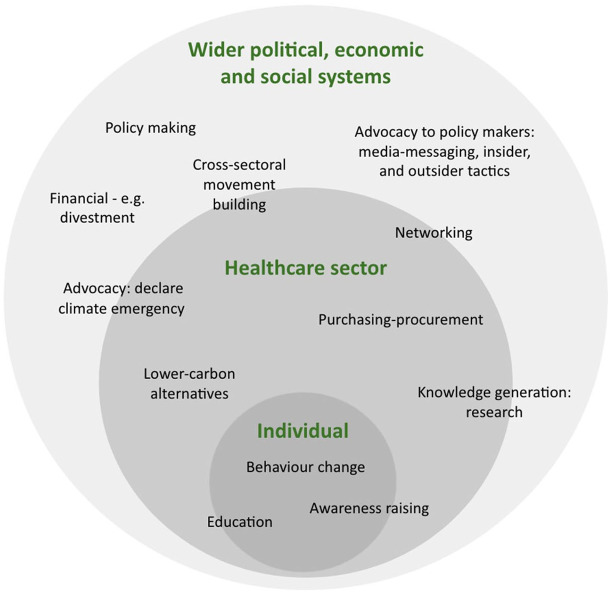
Domains and means to influence change

There are a range of organisations working across England, using different methodologies and targeting different stakeholders to influence action on climate change and health. The rapid rise in the number of organisations working on these themes over recent years shows increasing interest and opportunity: from a small number of fore-runner/early advocate organisations who worked in relative isolation on what was viewed as a ‘fringe’ issue, to representation today which spans academia, hospital trusts, the royal colleges, social movements, and specialised NHS bodies. These organisations hold different theories of change, which may be implicit or explicit. No one theory of change can be applied to this ‘climate change and health’ movement, and as such, this discussion explores different theories of change by delineating the movement based on the target *domain* of influence (individual vs sectoral vs systems change), and the *means* of change across these domains (Figure 5). While the broad movement around climate change is multifaceted and spans in focus from individual-level action to radical system or structural change, the climate change and health space is somewhat skewed towards actions at the individual and sectoral levels.

### Action at the individual level

Individualised actions to address the climate crisis aim to encourage individuals to change their lifestyle voluntarily to reduce their CO_2_ emissions. This includes promoting recycling or active travel, purchasing green and re-usable products, and consuming a vegetarian or vegan diet. The assumption is that if a sufficiently large number of people can be persuaded to change their behaviour, a large-scale reduction in emissions can be achieved. There is some tension arising from differing theories of change about whether the focus should be individual or structural change.

The world’s richest 10% produce around half of the world’s CO_2_ emissions, and someone from the richest 1% of the world’s population uses on average 175 times more carbon than someone from the bottom 10%. The NHS is responsible for 5.4% of the UK’s total carbon emissions. Given that this is the case, particular theories of change would posit that influencing the course on climate change will require cumulative individual behaviour and norm change within the populations and sectors that are most consumptive. Shifts in social norms can often underpin change by guiding individual behaviour; individual behaviours, in turn, influence social norms.^9^ This is the case with the phenomenon of ‘flight shame’, where domestic air travel in Sweden decreased by 15.4% per month between 2018 and 2019^10^ in response to a social norm shaming the environmentally harmful impact of flying. Flight shame is an example of ‘self-categorisation theory’;^11^ the phenomenon whereby individuals self-categorise as part of a group and produce behaviours associated with that group to signal membership (in this case, with the identification of being ‘environmentally conscious’).

For the climate change and health movement, individual-level action is represented by groups such as Eco Medics, who utilise social media to influence individual behaviour.^12^ However, achieving the substantial shift to bring about change at the speed required may not be met through individual action and normative change alone, nor is it feasible for vast sections of the population.^13^ This focus on individual action – as seen in the wider climate movement – is unsurprising. ‘Climate change helplessness’ or ‘climate anxiety’^14^ may lead to a focus on personal behaviour – empowering when faced with structural or systems-based approaches to change that may be perceived to be time-consuming, difficult to engage with, and overwhelming. The concept of ‘individual responsibility’^15^ is already pervasive in healthcare – for example taking an individual versus systems view on ‘lifestyle’-driven diseases, and there may be a sense that individuals can’t take action on a systems level, or would be hypocrites to try, until sufficient personal change has been made. Finally, given historical and present-day examples of health workers and institutions engaging in the political realm, action at the systems level may also be deemed by health workers to be too ‘political’, tying in to concerns of professional accountability, duties of care, and a professional respectability that is nominally ‘apolitical’.

### Action at the healthcare sector level

Climate change and health groups in the UK are working through and targeting stakeholders from across the NHS, including within their places of work, managerial structures, regulatory bodies, and Royal Colleges (Figure 2). There are a significant number of groups working at the hospital trust level (e.g. Greener Barts), through local general practice networks or directly in GP practices (e.g. Greener Practice), and to exert speciality-specific influence through Royal Colleges, advocacy organisations, or in local regions (e.g. the RCEM special interest group, GASP (Greener Anaesthesia and Sustainability Project), or ‘Sustainable Anaesthesia in Peninsula’ respectively). Many of these groups set their target stakeholders within the ‘health’ space, for example, influencing NHS procurement, other health workers, or Royal Colleges. Working to influence individual trusts or practices is an extension of the individualised theories of change outlined above which focus on individual behaviours over structural reform; however, given the significant contribution of the health sector to carbon emissions, these approaches may be ultimately impactful, especially if groups with similar targets across geographical regions or within the same speciality engage in cross-collaboration, skill-sharing, and lessons learnt, to reduce duplication of work, inefficiencies, and burn-out, and maximise chances of success.^16^

The benefits of a national health service mean that coordination and collaboration can be facilitated centrally. The Sustainable Development Unit was established in 2008, and the Greener NHS campaign was launched in 2020 to ‘build on the work of trusts, share ideas on how to reduce the impact [of climate change] on public health and the environment, save money and reach net carbon zero’.^17^ The formation of the these centralised initiatives means the UK health service is heralded as being one of the most progressive in the sustainable healthcare field, as the only healthcare system globally to have estimated its carbon footprint and set reduction targets.

#### Declaring a climate and ecological emergency

The growing number of healthcare organisations declaring a climate and ecological emergency (CEE) – including 10 hospital trusts since 2019 – is a product of the success of a mass movement towards climate action. Declaring a CEE can be an important step for an institution, particularly for those without a record of climate action. For members and organisations, it can be a direct way to ‘act within your sphere of influence’ to achieve tangible – and comfortable – goals. There are limitations to the effectiveness of this strategy, however. Declaring an emergency is merely a symbolic act unless followed up with further concrete action. For example, the Canadian government signed up to the expansion of an oil pipeline the day after becoming the second country to declare a climate emergency in June 2019.^18^ The vast majority of CEE declarations avoid being prescriptive about specific policies in order to be palatable to a wider range of the political spectrum. As such, the declaration of an emergency needs to be followed by a detailed plan of implementation. By declaring a climate emergency, health organisations publicly acknowledge the gravity of the crisis and realign their organisational goals in line with an overarching aim of cutting carbon emissions. If this is an introspective pursuit and the goal is to just act on institutional or specialty behaviour, the implied assumption is that other organisations will come on board with similar approaches, otherwise, the overall impact is negligible. However, the declaration of a CEE gives the institution the backing of its members to pursue broader advocacy: communicating with members and the public about the public health dimension of the climate crisis, and putting pressure on policy-makers through political advocacy, though it may still fall short of political discourse aimed at transforming public policy in the way necessary to meet the climate crisis.

#### Networking

There is an implied assumption that the early adopters of public statements such as declaring a CEE will be joined by other players in the ‘network’ – hospital trusts, royal colleges, and organisations – to achieve a critical mass and norm change across institutions. Utilising networking as a theory of change methodology draws from coalition theory,^19^ where coalitions come together by agreement over shared core beliefs about policies, and who can then explore and pursue multiple avenues for change – for example, by engaging in legal advocacy or working on changing public opinion – often simultaneously, to find a route that will bear fruit. A number of organisations exist to formally facilitate such networking and skill sharing, for example, the UK Health Alliance on Climate Change (UKHACC)^20^ – which connects established health organisations, and ‘Health Declares’,^21^ which connects regional and speciality groups – made up of members – through a framework of action to influence institutions providing healthcare (such as Trusts) as well as governing bodies (such as the Royal Colleges). In these examples, we note how groups operating at a similar ‘level’ of influence (e.g. member groups vs organisational governing bodies) seem to benefit from organisations that facilitate networking, but that networking seems relatively constrained to being *within* but not *across* these levels.

### Action at the systems level

Of the organisations identified, relatively few are focused on changing economic and political systems beyond the healthcare sector. Those that do may broadly share certain aims, for example, the need to reduce greenhouse gas emissions, but have differing views on how decarbonisation should be achieved, as well as at what speed. Many of these groups also differ greatly in the methods they employ to achieve their aims. Organisations exist on a spectrum between ‘incremental system change’ and ‘radical system change’, which maps to the tactics utilised, including ‘insider’ and ‘outsider’ approaches (Box 4).

**Box 4:** 

**Insider and outsider approaches** ^22^
Insider organisations work to influence and effect change inside political institutions, with engagement that is participatory and aimed at achieving cooperation. As such, insider approaches are more likely to call for incremental change, where demands are more aligned with political consensus and with the leadership within the healthcare community. Insider tactics include lobbying, expert information, official hearings, and other direct communication with decision makers. Outsiders, in contrast, work to effect social change from outside political institutions, often by challenging these institutions and their policies. This may be because they lack close links with policy-makers, or are reluctant to engage in direct contact with institutions in order to maintain a critical, oppositional role able to call for more radical change. Outsider strategies include demonstrations, petitions, civil disobedience, boycotts, media visibility, and other forms of communication and pressure in the public sphere. Although seemingly opposed, Insider and Outsider approaches can be complementary and have been integral to the success of a number of social movements: for example, outsider groups calling for more radical demands help shift the Overton window – the range of policies politically acceptable to the mainstream population at a given time – which facilitates the ‘soft power’ of insider groups to lobby for stronger policies.

#### Advocacy to policy makers: media-messaging

Relatively few organisations formally seek to reframe climate change as a public health issue in the public domain, though this may be a ‘side-effect’ of the work of research institutions and other campaigning organisations. When campaigners successfully articulate a political frame that ‘resonates’ with sufficient numbers of people in society, large-scale change is possible.^23^ Research suggests that broad sections of the population respond positively to taking action on climate change when the issue is presented through a public health framing: it generates support for efforts at mitigation and adaptation to climate change among groups who are unresponsive to its traditional presentation as an ‘environmental issue’,^24^ and in some cases has been cited as the most convincing argument to take action. From the 1990s onwards, the healthcare profession helped to reframe smoking in enclosed venues from being a matter of personal choice to being a public health concern, paving the way for the 2007 smoking ban in England. These approaches draw on a ‘messaging and frameworks’ theory of change, which understands that individuals develop different preferences based on how options are presented or framed; and ‘diffusion theory’,^19^ where policy makers are influenced by new ideas which have been accepted by a critical mass of the population, having been communicated by trusted messengers.

Healthcare professionals are among the most trusted professions;^25^ there is therefore scope for healthcare leaders to be persuasive advocates. However, few organisations were identified that included using the healthcare voice or messaging for the wider climate change movement in its aims or methodology. Doctors for Extinction Rebellion, a subgroup of the ‘Extinction Rebellion’ movement who have sought to make the connection between the climate crisis and public health ‘visible’ in this way, using ‘outsider’ tactics such as street action and stunts to gain media coverage. Medact members are building cross-sectoral collaboration through the campaign ‘Health for a Green New Deal’, which provides a public health framing for the creation of green jobs, offering a ‘health-voice’ to strategically build social pressure in support of key policies at both local and national levels of government.

#### Advocacy to policy makers: insider approaches and knowledge generation

Groups engaged in political action on climate change and health exhibit one of two broad political approaches for addressing the social determinants of health, as described by Dennis Raphael.^26^ The first is a ‘professionally-oriented’ approach that involves the dissemination of knowledge and advocacy by healthcare professionals with the aim of convincing policy-makers to enact health-supporting policies. This corresponds to what political scientists term a ‘pluralist’ understanding of the political process as relatively open and responsive to competing interest groups and guided by the quality of ideas in the public arena, and draws on the ‘policy window’^19^ theory of change whereby problems, policies and politics converge, and where policy options developed through research and publications have the opportunity to be adopted. These approaches generally require good relationships and reputations, both of which are generally afforded to and the remit of ‘insider’ organisations, who influence change by working directly with those with power to influence decision making.^19^ Such approaches are generally aligned with incremental system change, as utilised by organisations like the UKHACC, who uses its position as a network of established health organisations to exert sort power and influence on decision makers, with demands that are relatively in line with political consensus. There are nonetheless limitations to a professionally oriented approach focused purely on engaging policy-makers and other elite stakeholders with scientific findings. It may be that policy-makers are not receptive to these findings or that the prescribed policy solutions conflict with core tenets of their ideology. Alternatively, fossil fuel companies and other powerful corporate actors who benefit from the status quo may ‘veto’ any proposed change through the informal power they wield over the policy-making process.

#### Movement-building and ‘outsider’ advocacy

The second political approach for addressing the social determinants of health is a ‘movement-based’ one^26^ that mobilises collective political action as a means to confront power-holders and drive change. A movement-based approach aims to mobilise public opinion and shift social norms through action that takes place outside official institutions, and more often (though not exclusively) aligns with ‘outsider’ tactics, and with a more radical view on change. Such tactics have been used by groups such as Doctors for Extinction Rebellion, whose use of nonviolent direct action (NVDA) with varying degrees of success in creating ‘dissensus politics’,^27^ or the ‘positive effects of polarisation’,^28^ provokes those with power to clarify their position on a particular issue and shift popular opinion either in support of or in opposition to them. Other movement-based organisations – such as Medact – may also be more radical in their climate targets and are more likely to be ‘intersectional’, linking the climate crisis and policy demands to broader interconnected social and economic issues.

#### Financial systems and divestment

Actions demanding health institutions divest any holdings in the top 100 fossil fuel companies saw a degree of success in the mid 2010s and moved from being a relatively ‘outsider’ issue to a ‘norm’ adopted by professional institutions such as UKHACC and the BMJ. Presently, though there are active divestment campaigns targeting medical indemnity organisations, the number of health institution divestment campaigns has declined, and despite previous divestment campaigns – such as the 2015 Wellcome Trust divestment campaign led by Medact and the Guardian^29^ – institutions still maintain investments in fossil fuels at odds with their organisational priorities, which may be reflective of the ongoing dominance of the fossil fuel economy.

### Study limitations

A whole system mapping would ordinarily include groups who would impact a system even if not explicitly aiming to do so; however, we excluded groups not directly aiming their work at influencing climate change and health – such as those working on plant-based diets – from our analysis. Combining trust groups, Royal College groups, and educational institutions skewed our figures in terms of numerical values, though still hold weight in qualitative analysis. As with any research, there is potential influence from the authors. The majority of the authors of this article are active within the field of climate change and health, and as such, have their own potential biases and assumptions; however, we have attempted to mitigate for this by ensuring the representation of a number of different types of organisations in the authorship team, and by a process of self and collective reflection during the writing process.

## Conclusion

Ecosystem mapping the climate change and health movement in England has highlighted a number of key themes for consideration. Overall, there is a focus on individual level and sectoral change, over system change. For groups working at the local level – be it through CCGs, GP practices, specialities, and/or Royal Colleges – there may be benefit from better coordination, collaboration, and a degree of centralisation for certain tasks, which may be fulfilled by the Greener NHS as it becomes more established. Similarly, certain activities could focus on centralised policy change for expediency and impact: for example, lobbying NICE to introduce an ecological component to prescribing guidelines versus working to change the prescribing choices of GPs on a practice-by-practice basis. For organisations operating to influence change on a systems level, many unsurprisingly utilise the insider influence that is afforded to the health professions resulting from respectability and societal position. More could be made of the potential to utilise the healthcare professions voice for the climate movement more broadly – through the media, or to support in wider messaging to influence public opinion and policy – in light of the evidence that a public health framing on the climate works. A shortcoming of the ‘movement’ is that it may not see itself as such and thus not take steps to work in a coordinated manner. What remains is an amorphous, complex system of multiple, passionate players left exposed to the ‘tyranny of structurelessness’ – where an apparent lack of structure can result in unaccountable leadership. Recognising that there is value in working to influence change across various points in an ecosystem, and given the rapid boom of climate change and health organisations in recent years, there may be benefit in a mind-set shift within the climate change and health space in England: with more coordination and collaboration to reduce unnecessary work and duplication, better identify movement gaps, and lead to more cohesive outcomes.
